# Do LDE-Methotrexate lipid nanoparticles decrease allograft vasculopathy in rabbit transplanted heart ?

**DOI:** 10.1186/1749-8090-8-S1-O149

**Published:** 2013-09-11

**Authors:** N Stolf, A Fiorelli, D Lorenço, L Barbieri, E Tavares, R Maranhão

**Affiliations:** 1 Cardiovascular Surgery, HCFMUSP-INCOR São Paulo, Brazil; 2 Cardiovascular Surgery, Beneficencia Portuguesa São Paulo, Brazil

## Background

Cardiac allograft vasculopathy is the major fator limiting long term survival after heart transplantion. In a previus study we have shown that LDE binded with placitaxel decreases graft vasculopathy disease in rabbits. The objective of this study is to investigate the influence of LDE-Methotrexate nanoparticle in development of vasculopathy in rabbits tranplanted heart and expression profiles of cellular receptors, inflammatory mediators and metalloproteinases.

## Methods

Heterotopic heart transplantation in cervical region was performed in twenty white male rabbits divided in two groups:

1. LDE-Metotrexate group: 10 rabbits treated with intravenous methotrexate particle once a week.

2. Control group: 10 rabbits treated with 3 ml of intravenous saline solution once a week.

All animals were fed with food with 0.5% of cholesterol and receveid 10 mg/kg/day of cyclosporine. After six weeks of observation, the animals were sacrificed.

## Results

Inflammation evaluated by macrophage distribution was reduced in animals treated with metotrexate from 27.1% to 6.0% (p= 0.0020), compared to control group. Stenosis of coronary arteries had threefold decrease (p = 0.0010) in LDE-Methotrexate group.

Values of relative gene expression profiles of Low density lipoprotein receptor ; Low density lipoprotein receptor – related protein 1; IL-18; TNFα; VCAM1 (Vascular cell adhesion molecule-1) ; MCP-1 (Monocyte chemotactic protein-1) and MMP-12 (Matrix metalloproteinase 12 ) were significantly reduced (p< 0.05) in methotrexate group.

## Conclusion

Intravenous methotrexate with nanoparticules of LDE-Methotrexate reduced allograft vasculopathy in transplanted hearts and inflamation markers.

